# Supplementary material for: The structure, redox chemistry and motor neuron toxicity of heterodimeric zinc-deficient SOD1-implications for the toxic gain of function observed in ALS
Source: Neurobiol Dis. Author manuscript; Available in PMC 2026 Jan 2. (PMC12758609; doi:10.1016/j.nbd.2025.107189)
Supplement: 1 [file NIHMS2127910-supplement-1.pdf]

Supplemental Table 1. Source parameters for the 6545XT QTOF for native protein mass spectrometry.

**Agilent 6545XT AdvanceBio LC/Q-TOF**	
Source	Agilent Jet Stream
Dry Gas Temperature	180 °C
Dry Gas Flow	10 L/min
Nebulizer	30 psig
Sheath Gas Temperature	150 °C
Sheath Gas Flow	10 L/min
VCap	5000 V
Nozzle Voltage	2000 V
Fragmentor	300 V
Skimmer	220 V
Quad AMU	*m/z* 500
Mass Range	*m/z* 300–7000
Acquisition Rate	1.0 spectrum/s
Acquisition Mode	Positive, extended (*m/z* 10,000) mass range

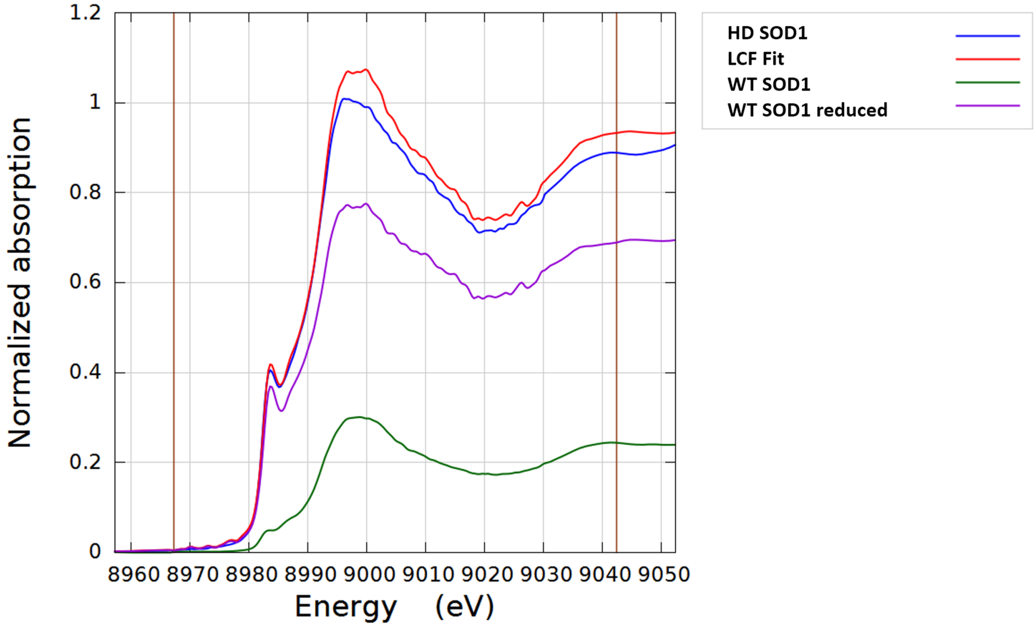

Figure S1. Linear combination fitting (LCF) to the D83S-C111S SOD1 heterodimer XANES data using WT and reduced SOD1 data as the fitting standards. LCF is based on XANES normalized μ(E) data region (E_0 -15, + 60 (eV); E_0 – the photoelectron energy threshold) marked by vertical lines.
